# Supplementary material for: A fault diagnosis method for rotating machinery components based on enhanced YOLO v8 and integrated attention mechanism
Source: PLoS One. 2025 Dec 12;20(12):e0338387. doi: 10.1371/journal.pone.0338387 (PMC12700396; doi:10.1371/journal.pone.0338387)
Supplement: Attachment — Submitted filename: PONE-D-25-42680-Review comments.docx [file pone.0338387.s001.docx]

**Review comments of Manuscript PONE-D-25-42680**

In response to the two major pain points in fault diagnosis of rotating machinery components— “easy loss of small-target fault features” and “insufficient deep feature extraction—” this paper proposes an improved scheme that embeds ODConv (Omni-dimensional Dynamic Convolution) and CBAM (Convolutional Block Attention Module) into the YOLO v8 framework (termed YOLO v8-C-OD). Comprehensive experimental validation was conducted on the CWRU, Paderborn, and self-built gearbox datasets. The overall methodology is clear, the workload is substantial, and the experimental results are persuasive.

However, there are still areas that need to be revised and supplemented in terms of the format and innovation of the article as follows:

1. I don't understand why the article requires images and manuscripts to be uploaded separately. Why don't authors insert images into the manuscript to make it easier for reviewers and readers to read and study?
2. The migration of the YOLO series object detection architecture to mechanical fault diagnosis is not without precedent. The authors need to define their contributions more precisely: is replacing the convolutional operators and attention modules alone sufficient to support the claim of a ‘novel fault diagnosis method’? To highlight the innovativeness of their paper, the authors need to provide further details.
3. The results are too perfect and lack statistical validation. Some of the results show a diagnostic accuracy of 100%, which is difficult to achieve in complex working conditions. Rather than simply providing the average accuracy rate, it is recommended that the authors supplement the mean and standard deviation of multiple experiments. Cross-validation or confidence intervals could also be added to improve the credibility of the results.
4. The ablation experiments in this paper are inadequate. Although there are some comparative experiments, the benefits of ODConv and CBAM have not been clearly distinguished. It is therefore recommended that the authors supplement the paper with explanations of each module's contribution.
5. Additionally, there are significant issues with the paper's formatting, citations and image resolution. For instance, the explanations of the symbols in equations 3, 4 and 6 are incomplete. It is recommended that the physical meanings of the variables are supplemented. The citation format is inconsistent. The resolution of Figures 8, 11 and 16 is slightly low. The authors are recommended to systematically revise these issues.

Addressing the issues outlined in the specific review comments would strengthen the manuscript and its suitability for publication in *PLOS ONE*.
