# Supplementary material for: A fault diagnosis method for rotating machinery components based on enhanced YOLO v8 and integrated attention mechanism
Source: PLoS One. 2025 Dec 12;20(12):e0338387. doi: 10.1371/journal.pone.0338387 (PMC12700396; doi:10.1371/journal.pone.0338387)
Supplement: Attachment — Submitted filename: Response to Reviewers.docx [file pone.0338387.s003.docx]

**Reviewer #1:**

We thank reviewer for all these valuable suggestions. We have corrected and improved the whole manuscript according to these suggestions point by point.

1. I don't understand why the article requires images and manuscripts to be uploaded separately. Why don't authors insert images into the manuscript to make it easier for reviewers and readers to read and study?

**Answer:** We thank reviewer for this comment, and sincerely thank you for your meticulous attention and valuable suggestions regarding the figure formatting in the manuscript. Due to the journal's suggestion that submitted manuscript files should not exceed 20MB, and the requirement that figures be uploaded as separate files, we uploaded the figures and text separately. We fully understand the inconvenience this may have caused you during the review process and sincerely apologize for this. We have incorporated all relevant figures into the corresponding locations within the main text as suggested, enabling you to intuitively reference the chart content while reviewing the textual logic.

2. The migration of the YOLO series object detection architecture to mechanical fault diagnosis is not without precedent. The authors need to define their contributions more precisely: is replacing the convolutional operators and attention modules alone sufficient to support the claim of a ‘novel fault diagnosis method’? To highlight the innovativeness of their paper, the authors need to provide further details.

**Answer:** We thank reviewer for this comment. This has prompted us to recognize more clearly the shortcomings in defining the paper's innovation.

(1) The point you raised—that “Transfer YOLO is not original”—is indeed the starting point we must confront. We do not pursue the ‘originality’ label, but rather aim to solidify the academic claim of a “novel fault diagnosis method” through rigorous details that make it both defensible and comprehensible. We can address this from three specific angles:

Regarding theoretical adaptability, Section 2.1 analyzed that while YOLO v8 enhances learning capacity by replacing C3 with C2f, subtle vibration fault features remain highly elusive and prone to omission during feature extraction—particularly evident in small object detection. Furthermore, Section 2.2 introduced the CBAM attention mechanism, which integrates channel and spatial attention to focus on critical information, reduce emphasis on secondary features, and improve fault feature extraction rates. Section 2.3 introduced ODConv, which dynamically adjusts convolution kernel weights. While maintaining low computational overhead and parameter count, it significantly enhances fault feature extraction capability, demonstrating outstanding performance in small object detection and fine-grained classification tasks. Theoretical derivation indicates that integrating CBAM and ODConv effectively improves YOLO v8's fault detection performance.

Experimental validation involves comparisons with classical methods (e.g., MTSTLF, DGNN) and similar transfer methods (e.g., CNN-HMM) on the same dataset. Comparative results demonstrate the superiority of the proposed method.

In terms of contribution quantification, we supplement Section 4.3 with ablation experiments. Using the controlled variable method, we validate that: introducing CBAM alone enhances feature focusing capability and improves model diagnostic accuracy; introducing ODConv alone optimizes weight allocation efficiency, boosts model diagnostic accuracy, and reduces computational complexity; while the synergistic effect of both achieves a performance leap.

(2) We further validate the contributions of CBAM and ODConv modules to the model, as well as their synergistic effect in achieving a performance leap for the YOLO v8 model, through supplementary ablation experiments in Section 4.3. The specific supplementary experimental content is as follows:

The ablation experiment results are shown in Table 8, conducted under three dataset conditions. Table 8 shows that CBAM improved classification accuracy by 0.11%, 0.7%, and 1.25% across different datasets. Meanwhile, the parameter GFLOPs (gigaflops per second) indicates that ODconv not only enhanced model performance (increases of 0.1%, 1.1%, and 1%) but also significantly reduced computational complexity. This demonstrates that both CBAM and ODconv effectively enhance model classification performance, with ODconv additionally offering the advantage of reduced computational complexity. When synergistically applied, the model achieves optimal classification accuracy, fully validating their collaborative effect.

Table 8. Ablation experiment results for model classification accuracy across different datasets.

| Dataset | CBAM | ODconv | Diagnostic average accuracy（%） | GFLOPs | epoch |
| --- | --- | --- | --- | --- | --- |
| CWRU Dataset | × | × | 99.80 | 3.3 | 50 |
|  | √ | × | 99.91 | 3.3 | 50 |
|  | × | √ | 99.90 | 2.6 | 50 |
|  | **√** | **√** | **100** | **2.6** | 50 |
| Paderborn University Dataset | × | × | 96.50 | 3.3 | 80 |
|  | √ | × | 97.20 | 3.3 | 80 |
|  | × | √ | 97.60 | 2.6 | 80 |
|  | **√** | **√** | **98.30** | **2.6** | **80** |
| Gearbox Dataset | × | × | 97.50 | 3.3 | 100 |
|  | √ | × | 98.75 | 3.3 | 100 |
|  | × | √ | 98.50 | 2.6 | 100 |
|  | **√** | **√** | **99.75** | **2.6** | **100** |

3. The results are too perfect and lack statistical validation. Some of the results show a diagnostic accuracy of 100%, which is difficult to achieve in complex working conditions. Rather than simply providing the average accuracy rate, it is recommended that the authors supplement the mean and standard deviation of multiple experiments. Cross-validation or confidence intervals could also be added to improve the credibility of the results.

**Answer:** We thank reviewer for this comment. The challenge you highlighted—that “100% accuracy is difficult to achieve under complex operating conditions”—is precisely the reality we must confront. We fully acknowledge this suggestion and explicitly state that the 100% fault diagnosis rate reported for the Western Reserve University dataset is based solely on the current experimental test set results, which inherently lack validation for operational condition generalization. Accordingly, following your recommendation, we have supplemented Section 4.3 with statistical data from multiple repeated experiments conducted after the ablation tests. Additionally, we have introduced a cross-validation mechanism to redistribute the dataset for training and testing, thereby validating the model's performance from both statistical stability and generalization capability perspectives. The specific modifications are as follows:

Based on the original YOLO v8 model, this section evaluates the specific impact of the CBAM and ODconv components on model classification accuracy through ablation experiments. Consistent with previous experimental settings, each set of experiments was repeated 10 times to ensure accuracy. The mean, standard deviation, and relevant experimental data for each set of experiments conducted on different datasets using the proposed model are listed in Table 7. The fluctuation range of the model's fault diagnosis accuracy was controlled within ±0.75, indicating the model's stability across different datasets.

Table 7. Diagnostic accuracy, mean, and standard deviation of the YOLO v8-C-OD model across 10 experiments.

| Method | Dataset | Diagnostic Accuracy Rate per Experiment（%） | | | | | | | | | | Mean（%） | Standard deviation |
| --- | --- | --- | --- | --- | --- | --- | --- | --- | --- | --- | --- | --- | --- |
|  |  | 1 | 2 | 3 | 4 | 5 | 6 | 7 | 8 | 9 | 10 |  |  |
| YOLO v8-C-OD | CWRU Dataset | 100 | 100 | 100 | 100 | 100 | 100 | 100 | 100 | 100 | 100 | 100 | 0 |
|  | Paderborn University Dataset | 98.3 | 98.3 | 98.3 | 98.3 | 97.5 | 98.3 | 99.1 | 98.3 | 98.3 | 98.3 | 98.3 | 0.36 |
|  | Gearbox Dataset | 100 | 100 | 100 | 100 | 100 | 100 | 100 | 100 | 97.5 | 100 | 99.75 | 0.75 |

To further validate the model's accuracy and stability, we designed a cross-validation testing scheme. During implementation, the following operations were performed on different datasets: a number of time-frequency image samples equal to the test set were randomly sampled from each training set, and these samples were swapped with corresponding samples in the test set. After data reorganization, the model training and validation process was re-executed. The final cross-validation results are detailed in Table 9.

Table 9. Cross-validation test results of the YOLO v8-C-OD model across different datasets.

| Dataset | Average accuracy (%) | Average number of training iteration steps/times |
| --- | --- | --- |
| CWRU Dataset | 99.93 | 50 |
| Paderborn University Dataset | 98.60 | 80 |
| Gearbox Dataset | 99.50 | 100 |

Analysis of the experimental data reveals that the cross-validation results exhibit high consistency with the previous experimental outcomes. Specifically, across multiple datasets, the fluctuation range of fault diagnosis accuracy is consistently controlled within ±0.3%. This result indirectly confirms the robustness of the model architecture while validating the reproducibility of the experimental workflow and the reliability of the outcomes. Through systematic data permutation verification, we have both eliminated the random errors inherent in single-run experiments and ensured the model's generalization capability across different data distributions, providing a reliable theoretical foundation for subsequent practical applications.

4. The ablation experiments in this paper are inadequate. Although there are some comparative experiments, the benefits of ODConv and CBAM have not been clearly distinguished. It is therefore recommended that the authors supplement the paper with explanations of each module's contribution.

**Answer:** We thank reviewer for this comment. This has deepened our understanding of the critical importance of distinguishing module contributions in ablation experiments. The point you raised—that “the advantages of ODConv and CBAM were not clearly differentiated”—indeed represents a key shortcoming in the original experimental design. While our focus was on validating the effectiveness of module combinations, we failed to sufficiently deconstruct the independent contribution mechanisms of individual modules.

To address this, we have conducted a dedicated supplementary ablation study in Section 4.3 to observe specific changes in model performance when individual modules are removed. Quantitative comparisons confirm that both CBAM and ODConv enhance the fault detection accuracy of the YOLO v8 model, while ODConv effectively reduces computational complexity. Their synergistic interaction achieves a significant performance leap. The specific modifications are as follows:

The ablation experiment results are shown in Table 8, conducted under three dataset conditions. Table 8 shows that CBAM improved classification accuracy by 0.11%, 0.7%, and 1.25% across different datasets. Meanwhile, the parameter GFLOPs (gigaflops per second) indicates that ODconv not only enhanced model performance (increases of 0.1%, 1.1%, and 1%) but also significantly reduced computational complexity. This demonstrates that both CBAM and ODconv effectively enhance model classification performance, with ODconv additionally offering the advantage of reduced computational complexity. When synergistically applied, the model achieves optimal classification accuracy, fully validating their collaborative effect.

Table 8. Ablation experiment results for model classification accuracy across different datasets.

| Dataset | CBAM | ODconv | Diagnostic average accuracy（%） | GFLOPs | epoch |
| --- | --- | --- | --- | --- | --- |
| CWRU Dataset | × | × | 99.80 | 3.3 | 50 |
|  | √ | × | 99.91 | 3.3 | 50 |
|  | × | √ | 99.90 | 2.6 | 50 |
|  | **√** | **√** | **100** | **2.6** | 50 |
| Paderborn University Dataset | × | × | 96.50 | 3.3 | 80 |
|  | √ | × | 97.20 | 3.3 | 80 |
|  | × | √ | 97.60 | 2.6 | 80 |
|  | **√** | **√** | **98.30** | **2.6** | **80** |
| Gearbox Dataset | × | × | 97.50 | 3.3 | 100 |
|  | √ | × | 98.75 | 3.3 | 100 |
|  | × | √ | 98.50 | 2.6 | 100 |
|  | **√** | **√** | **99.75** | **2.6** | **100** |

These supplementary experiments not only address your concerns regarding the “contribution mechanism” but also provide us with a clearer understanding of the interaction principles between modules. We fully recognize that the rigor of scientific research lies in the pursuit and verification of details. Therefore, we earnestly request reviewers to continue pointing out our shortcomings and assist us in further refining this study.

5. Additionally, there are significant issues with the paper's formatting, citations and image resolution. For instance, the explanations of the symbols in equations 3, 4 and 6 are incomplete. It is recommended that the physical meanings of the variables are supplemented. The citation format is inconsistent. The resolution of Figures 8, 11 and 16 is slightly low. The authors are recommended to systematically revise these issues.

**Answer:** We thank reviewer for this comment. Your keen observation regarding the missing symbol explanations in Equations 3, 4, and 6, the inconsistencies in citation formatting you pointed out, and the issues with insufficient resolution in Figures 8, 11, and 16 are invaluable to us.

(1) Improvements to the Explanation of Equation Symbols: We have reviewed each variable symbol individually and added corresponding explanatory notes for each one. The specific modifications are as follows:

$W_{0}$ and $W_{1}$ are the weight of two MLP layers;

$A\text{v}gPool$—Average Pooling;

$M\text{ax}Pool$—Maximum pooling;

$W_{\phi} (a,b)$— The result of the wavelet transform (Wavelet coefficient);

$x(t)$— Signal to be analyzed.

(2) Regarding the standardization of citation formats: This was indeed due to our oversight. We meticulously reviewed each entry in the reference list, strictly reformatted them according to the journal's requirements, and corrected all inconsistencies in author name capitalization and journal abbreviations. We have made the following modifications:

For example：

Reference [3]

[3] Zhao Y, Wang J, Li X, Peng G, Yang Z. Extended least squares support vector machine with applications to fault diagnosis of aircraft engine. Isa Transactions. 2020;97:189-201.

Reference [6]

[6] Chen P. Review of fault diagnosis methods for rolling bearings based on vibration signals. Bearings. 2022(06):1-6.

(3) Regarding the enhancement of image resolution: We have completely remastered these images and increased their resolution to over 600 dpi to ensure precise visibility in video footage. Additionally, the corresponding images have been embedded at their respective locations within the main text of the paper.

**Reviewer #2:**

We thank reviewer for all these valuable suggestions. We have corrected and improved the whole manuscript according to these suggestions point by point.

1. Please modify the format of the paper according to the submission requirements of PLOS ONE, such as aligning the paragraphs at both ends.

**Answer:** We thank reviewer for this comment. This provides important guidance for enhancing the academic rigor and readability of our paper. Regarding the formatting issues you pointed out, we have thoroughly reviewed and revised the manuscript in strict accordance with PLOS ONE's submission requirements, including aligning paragraphs to both margins and adjusting line spacing.

2. Could the author explain why the YOLO v8 model has already achieved high prediction accuracy in Tables 2, 5, and 6 for equipment fault detection, but the YOLO v8-C-OD fault diagnosis model is still proposed? What advantages does the proposed model have?

**Answer:** We thank reviewer for this comment. This prompts us to reflect more deeply on the necessity of model refinement.

(1) The observation that “YOLO v8 achieves high accuracy in Tables 2, 5, and 6” indeed served as the starting point for our work. However, these very datasets revealed the limitations of the original model—while demonstrating excellent overall performance, it still showed room for improvement in complex scenarios such as detecting minor vibration faults and extracting features from small objects.

Specifically, while the original YOLO v8 model achieves high-precision diagnostics under standard conditions, its C2f architecture risks “over-smoothing” during feature extraction when confronting the “feature concealment” challenge common in industrial settings—such as the faint vibration signals generated by early-stage equipment failures—thereby weakening critical fault signatures. This is precisely the core motivation behind our proposed YOLO v8-C-OD model: integrating the CBAM attention mechanism with the ODConv dynamic convolution module to achieve a significant leap in overall performance. We do not dismiss the value of the original model but aim to make the fault diagnosis model more aligned with industrial practical needs through this “precision enhancement.” We supplemented Section 4.3 with ablation experiments and quantitatively validated the independent contributions and synergistic effects of each module.

(2) The new model's advantages manifest across three dimensions: First, CBAM precisely localizes fault feature regions through dual channel-spatial attention, reducing focus on secondary features and enhancing fault feature extraction rates; Second, ODConv dynamically adjusts convolution kernel weights to significantly enhance small object detection capabilities while maintaining low computational overhead. Third, the synergistic interaction between these two components forms a positive feedback loop of “feature enhancement-weight adaptation,” substantially improving the model's fault detection performance across operating conditions and in small-object scenarios. We kindly request reviewers to continue focusing on refining these details to help us enhance the practical guidance of this research. The specific modifications are as follows:

The ablation experiment results are shown in Table 8, conducted under three dataset conditions. Table 8 shows that CBAM improved classification accuracy by 0.11%, 0.7%, and 1.25% across different datasets. Meanwhile, the parameter GFLOPs (gigaflops per second) indicates that ODconv not only enhanced model performance (increases of 0.1%, 1.1%, and 1%) but also significantly reduced computational complexity. This demonstrates that both CBAM and ODconv effectively enhance model classification performance, with ODconv additionally offering the advantage of reduced computational complexity. When synergistically applied, the model achieves optimal classification accuracy, fully validating their collaborative effect.

Table 8. Ablation experiment results for model classification accuracy across different datasets.

| Dataset | CBAM | ODconv | Diagnostic average accuracy（%） | GFLOPs | epoch |
| --- | --- | --- | --- | --- | --- |
| CWRU Dataset | × | × | 99.80 | 3.3 | 50 |
|  | √ | × | 99.91 | 3.3 | 50 |
|  | × | √ | 99.90 | 2.6 | 50 |
|  | **√** | **√** | **100** | **2.6** | 50 |
| Paderborn University Dataset | × | × | 96.50 | 3.3 | 80 |
|  | √ | × | 97.20 | 3.3 | 80 |
|  | × | √ | 97.60 | 2.6 | 80 |
|  | **√** | **√** | **98.30** | **2.6** | **80** |
| Gearbox Dataset | × | × | 97.50 | 3.3 | 100 |
|  | √ | × | 98.75 | 3.3 | 100 |
|  | × | √ | 98.50 | 2.6 | 100 |
|  | **√** | **√** | **99.75** | **2.6** | **100** |

3. Is the sample set in the YOLO v8-C-OD fault diagnosis model sufficient, and is the data collection method scientific?

**Answer:** We appreciate the reviewers' meticulous attention to the selection and collection methods of the dataset. This rigor has deepened our understanding that “data quality is the lifeline of a model.”

(1) In experimental validation, sample adequacy directly impacts a model's generalization capability, primarily manifested as the balancing act between overfitting and underfitting. Overfitting refers to a model exhibiting “false high performance” on the training set—excessive adaptation to known samples leads to a sharp decline in test set performance, typically characterized by inflated training accuracy and significantly reduced test accuracy. Underfitting manifests as the model failing to fully learn the fundamental patterns in the training data, resulting in poor performance on both the training and test sets. Both phenomena are direct reflections of insufficient sample size or imbalanced data distribution. For fault detection classification tasks, model evaluation metrics typically include accuracy, metrics/accuracy_top1, and metrics/accuracy_Top5.

This paper employs a dual validation approach using accuracy and confusion matrices: across the three datasets utilized, the model achieved test set accuracy exceeding 98.3%. Compared with other fault diagnosis models, this demonstrates the superior performance of the proposed model in fault diagnosis. Taking the West China University dataset as an example (Figure 12), Comparing the trends of the two curves in the metrics/accuracy_top1 and metrics/accuracy_Top5 plots indicates that diagnostic accuracy improves synchronously across both training and validation sets. This confirms the model exhibits neither overfitting nor underfitting, while also demonstrating that it has fully learned the essential features of various faults.

(a) confusion matrix normalized	(b) results

**Fig 12. Bearing Fault Diagnosis Confusion Matrix.**

Additionally, this paper supplements the cross-validation experiments. The accuracy metrics and accuracy_top1 obtained through cross-validation show high consistency with the original experimental results, with fluctuations remaining within 0.3%. This demonstrates the stability of model performance under the datasets used in this paper and simultaneously validates the adequacy of the sample size. The specific modifications are as follows:

To further validate the model's accuracy and stability, we designed a cross-validation testing scheme. During implementation, the following operations were performed on different datasets: a number of time-frequency image samples equal to the test set were randomly sampled from each training set, and these samples were swapped with corresponding samples in the test set. After data reorganization, the model training and validation process was re-executed. The final cross-validation results are detailed in Table 9.

Table 9. Cross-validation test results of the YOLO v8-C-OD model across different datasets.

| Dataset | Average accuracy (%) | Average number of training iteration steps/times |
| --- | --- | --- |
| CWRU Dataset | 99.93 | 50 |
| Paderborn University Dataset | 98.60 | 80 |
| Gearbox Dataset | 99.50 | 100 |

Analysis of the experimental data reveals that the cross-validation results exhibit high consistency with the previous experimental outcomes. Specifically, across multiple datasets, the fluctuation range of fault diagnosis accuracy is consistently controlled within ±0.3%. This result indirectly confirms the robustness of the model architecture while validating the reproducibility of the experimental workflow and the reliability of the outcomes. Through systematic data permutation verification, we have both eliminated the random errors inherent in single-run experiments and ensured the model's generalization capability across different data distributions, providing a reliable theoretical foundation for subsequent practical applications.

(2) This study constructed an experimental sample repository using multiple datasets, including two bearing failure datasets and one gearbox failure dataset. Both the CWRU and Paderborn University bearing datasets employed standardized acceleration sensor acquisition protocols, undergoing rigorous amplitude calibration and frequency response testing to ensure reliable signal quality. Due to their authority, data quality, scenario coverage, and ease of use, these datasets have become the “cornerstone datasets” for bearing fault diagnosis research. The Qianpeng Gearbox Dataset originates from real industrial scenarios, capturing four types of faults—including gear pitting and tooth breakage—under heavy-load, variable-speed, and high-temperature conditions. Its industrial context, practical utility, scenario coverage, ease of use, and broad applicability make it a vital resource for gearbox fault diagnosis. Whether for academic research, algorithm development, or industrial applications, these datasets provide reliable data support and standardized evaluation platforms, effectively reproducing the complex vibration characteristics of rotating machinery components under demanding operating conditions.

Additionally, in Section 4.1.2 of the article, we have supplemented and revised the basis for dataset division, further demonstrating the rigor and scientific validity of the data collection in this study. The specific modifications are as follows:

To clearly extract periodic fault features in time-frequency analysis, the data window must cover at least 5–10 complete fault impact cycles, providing sufficient feature learning space for subsequent time-frequency transformations or deep learning models. With a sampling frequency of 12 kHz and a total of 120,000 data points, 5 cycles correspond to approximately 674 data points, while 10 cycles correspond to approximately 1,348 points. This paper selects a sample length of 1000 points, covering approximately 7.2 fault cycles. This length offers advantages over longer samples like 2048 or 4096 in terms of GPU memory usage and computational efficiency, while aligning with industrial sliding window design principles (where sample length is typically 1/5 to 1/3 of the signal variation period, with 1000 points falling within this optimal range). The core data augmentation strategy employs a 50% overlap rate (where adjacent windows overlap by 500 points). This approach significantly enhances the robustness and stability of the features learned by the model by preventing fault impact events from being entirely missed by all windows. Simultaneously, it effectively expands the training dataset size, alleviating the deep learning model's demand for large data volumes. Ultimately, this achieves dual optimization: ensuring the completeness of feature extraction while enhancing the efficiency of model training.

4. Has the proposed YOLO v8-C-OD fault diagnosis model been used with self-collected data to predict faults in rotating mechanical components, to verify the accuracy and reliability of the proposed model? It is suggested to add actual cases or field test data.

**Answer:** We thank reviewer for this comment. The practical validation issues you raised regarding the model are indeed a key focus of our current research. We sincerely clarify that at this stage, the YOLO v8-C-OD fault diagnosis model has not yet been directly applied to self-collected rotating machinery component data or field testing scenarios. Therefore, we are currently unable to provide fault prediction cases or field test data from actual industrial environments to validate the model's accuracy and reliability.

This situation primarily stems from objective limitations inherent to the research phase: data collection requires deep collaboration with industrial sites, involving coordination for equipment shutdowns, synchronized deployment of multiple sensors, and signal acquisition and annotation under complex operating conditions. These processes necessitate extended preparation cycles and cross-departmental cooperation. Our current focus remains on optimizing the model algorithm and validating it in laboratory settings, completing foundational performance testing. Specifically, utilizing the gearbox fault dataset from Jiangsu Qianpeng Diagnostic Engineering Co., Ltd. (noted for its industrial background, data utility, scenario coverage, ease of use, and broad applicability) has effectively replicated real-world gearbox faults in industrial scenarios, ensuring the model meets expected performance metrics across various standardized datasets.

We fully appreciate the reviewers' concern regarding the model's practical industrial value, which aligns precisely with our core future research direction. Moving forward, we will prioritize the following initiatives: First, collaborating with industrial partners to establish a rotating machinery fault diagnosis testing platform for collecting multimodal data (vibration, sound, etc.) under real-world operating conditions; Second, designing rigorous field testing protocols to validate the model's fault prediction capabilities during actual equipment operation; Third, we will establish a quantifiable evaluation system to comprehensively assess the model's performance in real-world scenarios using metrics such as confusion matrices and accuracy rates. These steps will ensure the model ultimately withstands the rigors of industrial environments, effectively serving the fault prediction and health management of rotating machinery.

We fully recognize that only models validated in real industrial settings can demonstrate their true value. While current limitations exist, we have formulated a clear roadmap for subsequent work and will rigorously advance each validation task. We extend our gratitude once more to the reviewers for their guidance, which has clarified critical gaps in our research and strengthened our commitment to enhancing model reliability through practical validation.
